# Glycogen Synthase Kinase 3 Beta (GSK3β) Phosphorylates the RNAase III Enzyme Drosha at S300 and S302

**DOI:** 10.1371/journal.pone.0020391

**Published:** 2011-06-03

**Authors:** Xiaoli Tang, Ming Li, Lynne Tucker, Bharat Ramratnam

**Affiliations:** Department of Medicine, Warren Alpert Medical School of Brown University, Providence, Rhode Island, United States of America; Institut National de la Santé et de la Recherche Médicale, France

## Abstract

The canonical microRNA (miRNA) pathway commences with the enzymatic cleavage of the primary gene transcript (pri-miRNA) by the RNAase III enzyme Drosha in the nucleus into shorter pre-miRNA species that are subsequently exported to the cytoplasm for further processing into shorter, mature miRNA molecules. Using a series of reporter constructs, we have previously demonstrated that phosphorylation of Drosha at Ser 300 and 302 was required for its nuclear localization. Here, we identify GSK3β as the culprit kinase. We demonstrate that Drosha is unable to selectively localize to the nucleus in cells deficient in GSK3β. These findings expand the substrate base of GSK3β to include a central component of the miRNA biogenesis pathway.

## Introduction

MicroRNAs (miRNAs) are a class of endogenous nonprotein-coding small RNAs of ∼22 nucleotides in length that impact gene expression by sequence specific interaction with homologous mRNA leading to direct degradation or translational inhibition [1]–[3]. One specific miRNA may inhibit many target genes and one specific gene may be regulated by more than one miRNA. For example, both miRNA-125 a and b, the genes of which are located on different chromosomes, target the p53 protein as both miRNAs harbor similar seed sequences that share similarity to the p53 3′UTR [4],[5]. MiRNAs play increasingly recognized roles in several basic processes including cell signal transduction, tumorigenesis, tumor invasion and metastasis, stem cell renewal, immune function, apoptosis and reaction to stress [6]–[10]. The human genome is studded with some 1048 miRNA genes (miRbase; Release September 2010). The vast majority of miRNA genes are thought to be under the control of RNAP II with others being recently identified as substrates of RNAP III [11],[12]. Irrespectively, miRNA genes are initially transcribed to yield a primary, long transcript that undergoes successive processing in both the nucleus and cytoplasm. Nuclear processing is mediated by the RNase III enzyme Drosha to generate precursor miRNAs (pre-miRNAs) of ∼70 nucleotides in length. Pre-miRNAs are subsequently transported to the cytoplasm by export 5-Ran-GTP where they are cleaved by the RNase III enzyme Dicer to generate mature miRNAs [1]–[3]. Investigation into the molecular mechanisms of miRNA biogenesis at the transcriptional and translational levels has been intensively pursued [12]–[15].

Drosha plays a central role in miRNA biogenesis and recent work suggests that its expression level directly influences clinical outcomes in malignant disease (e.g. ovarian cancer) thus underlying the importance of better understanding mechanisms that impact Drosha expression and function [16]–[19]. The molecular anatomy of Drosha allows its dissection into two broad functional domains: a C-terminus that harbors enzymatic activity and an N-terminus that contains motifs for its nuclear localization [20],[21]. In our previous work, we focused on the N-terminal region of Drosha which was known to harbor a nuclear localization signal [20]. Using a series of reporter constructs of progressively shorter Drosha protein sequence, we narrowed down the NLS to aa270–390. Mass spectrometric analysis, mutagenesis and functional assays revealed that phosphorylation of Drosha at Ser 300 and 302 was required for its correct nuclear localization [22]. Here, we directly identify GSK3β as the culprit kinase.

## Methods

### Cell culture and transfection

HeLa cells were grown in Eagle's Minimum Essential Medium supplemented with 10% fetal bovine serum, 2 mM L-glutamine and non-essential amino acids. Cells were trypsinized and reseeded in culture plates one day before transfection. HeLa cells were transfected with Lipofectamine 2000 when cell confluency was ∼70%. Mouse Embryonic Fibroblast (MEF) cells were cultured in Dulbecco's modified Eagle's medium (Invitrogen, Carlsbad, CA, USA) with 10% fetal bovine serum (Thermo Scientific), 2 mM l-glutamine and non-essential amino acids (Invitrogen). Cells were trypsinized and reseeded in culture plates 1 day before transfection. Transfection was performed with Lipofectamine LTX and Plus reagent (Invitrogen) when cell confluency was ∼60%.

### Western blotting

Cell lysates (100 µg protein each) were separated by 4–12% SDS–PAGE electrophoresis and electroblotted to nitrocellulose membrane (Bio-Rad). Blotted membranes were probed with their respective primary antibodies rotating at 4°C overnight. The membranes were washed three times in TBST buffer and probed with secondary antibody (Alexa Fluor 680 goat anti-rabbit IgG or IRDye800-conjugated Affinity Purified Anti-Mouse IgG, respectively) at room temperature for 1 h. Membranes were then washed three times in TBST buffer and direct infrared fluorescence detection was performed with a Licor Odyssey® Infrared Imaging System [23].

### *In vitro* kinase assays

GSK3β−/− MEF cells were transfected with empty vector, GFP-Drosha or GFP-DroshaS300A/S302A, respectively. Forty eight hours post-transfection, cell lysates were prepared with IP/Lysis buffer (Pierce). Sepharose beads conjugated with polyclonal antibody against GFP (Abcam) were incubated with respective cell lysate at 4°C overnight. After three cycles of washing and centrifugation, the precipitates were used for an *in vitro* kinase assay using ^32^P-ATP and pure enzyme GSK3β (New England Biolabs) according to the manufacturer's instructions. The reaction products were resolved by 4–12% SDS-PAGE gel and electroblotted to nitrocellulose membrane (Bio-Rad). The blotted membrane was exposed to X-ray film to visualize the phosphorylated Drosha. A synthesized peptide (Abgent, San Diego, CA) harboring the SPSLERS motif with flanking amino acids (H-RERHRHRDNRRSPSLERSYKKEYKR-CONH2) was also used for an *in vitro* kinase assay. The reaction mixture which consisting of peptide and reagents described above was dot blotted to a nitrocellulose membrane directly and washed three times with TBST before exposing to X-ray film.

### Confocal fluorescent imaging

HeLa cells were transfected with a construct encoding GFP-Drosha using Lipofectamine 2000. Six hours post-transfection, the cells were treated for 20 hours with various kinase inhibitors as indicated including JNK inhibitor Sp600125 (A.G. Scientific), p38 MAPK inhibitor SB203580 (Sigma-Aldrich), CDK5 inhibitor roscovitine (Sigma-Aldrich), MEK inhibitor U0126 (Sigma-Aldrich), PKA inhibitor H-89 (BioVision) and GSK3β inhibitor CHIR99021 (BioVision). MEF cells were transfected with constructs expressing GFP, GFP-Drosha or mutated Drosha, respectively. To determine whether GSK3β expression restores Drosha nuclear localization in GSK3β^−/−^ MEF cells, the latter were transfected with GFP-Drosha or GFP-Drosha and GSK3β expression construct (Adgene). Twenty-four hours post-transfection, the cells were trypsinized and reseeded at 1∶10 dilution. The cells were incubated for another 24 h. Hoechst 33342 (final concentration 1 µg/ml) was added to cell culture 30 min before confocal fluorescent imaging which was performed with a LeicaTCS SP2 AOBS confocal laser microscope [22].

## Results

### GSK3β emerges as a likely candidate

**Figure 1:**
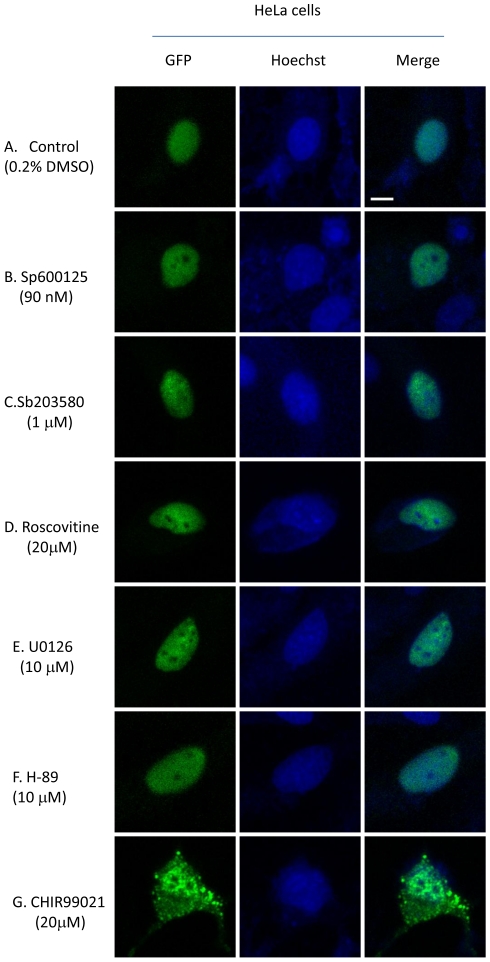
Effects of various kinase inhibitors on Drosha localization. HeLa cells were transfected with GFP-Drosha. Six hours post-transfection, the cells were treated with various kinase inhibitors as indicated for 20 hours. Thirty minutes before performing confocal imaging, 1 µg/ml Hoechst 33342 was added to the cells to stain the nuclei. A. Solvent control; B. JNK inhibitor Sp600125; C. p38 MAPK inhibitor SB 203580; D. CDK5 inhibitor roscovitine; E. MEK inhibitor U0126; F. PKA inhibitor H-89; G. GSK3β inhibitor CHIR99021. Only inhibition of GSK3β disrupted the nuclear localization of Drosha. Bar indicates 10 micrometers.

**Figure 2:**
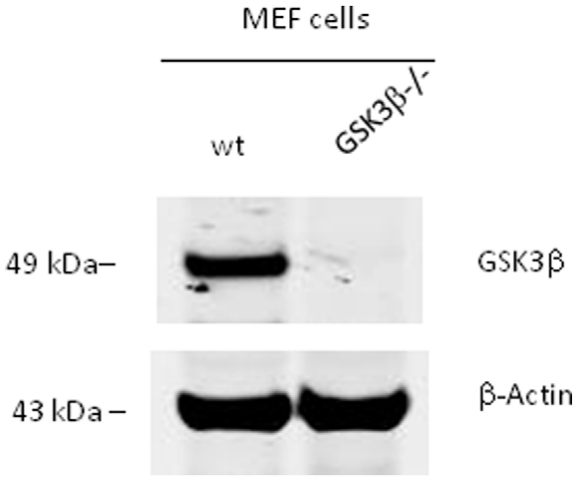
Expression levels of GSK3β protein in WT and KO MEF cells. We confirmed GSK3β protein expression in wild type MEF cells and its absence in GSK3β knockout MEF cells by western blot. β-Actin was used as a protein loading control.

**Figure 3:**
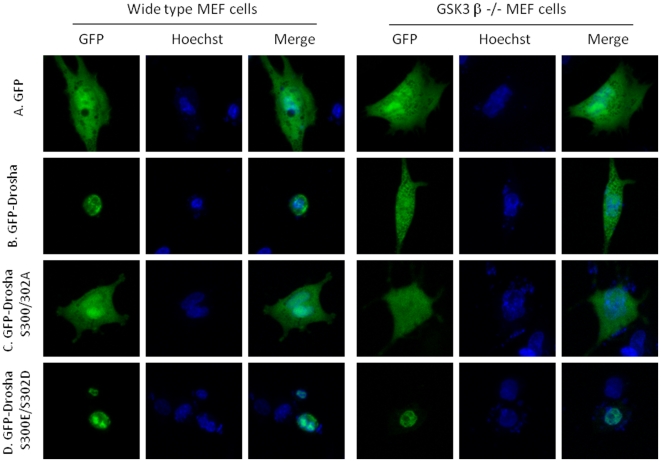
Phosphorylated Drosha localizes in the nucleus. A. GFP vector alone, showing diffuse expression pattern in both GSK3β wt and knockout MEF cells; B. GFP-Drosha localizes in the nuclei in GSK3β wt MEF cells but shows a diffuse expression pattern in GSK3β knockout MEF cells; C. Phosphorylation-deficient GFP-DroshaS300A/S302A diffusely expresses in both GSK3β wt and knockout MEF cells; D. Phosphorylation mimetic GFP-Drosha S300E/S302D localizes in the nuclei in both GSK3β wt and knockout MEF cells.

To identify the putative kinase(s) involved in phosphorylating Drosha at Ser 300/302, we first mined Drosha sequence using web based kinase prediction algorithms (e.g. KinasePhos 2.0: http://kinasephos2.mbc.nctu.edu.tw; PPSP system: http://ppsp.biocuckoo.org) and all identified GSK3β as the putative kinase among other candidates. To test the veracity of these bioinformatic predictions we first noted cellular localization patterns of a Drosha-GFP reporter construct in the presence of the various kinase inhibitors. With the exception of CHIR99021 (GSKβ inhibitor), treatment with all other inhibitors did not lead to cytoplasmic localization of Drosha. In contrast, treatment with CHIR99021 led to cytoplasmic localization, thus providing preliminary foundation for further investigation (Figure 1). Our interest in GSK3β was further increased by noting the presence of a classic GSK3β phosphorylation motif in Drosha comprised of Ser-Pro-Ser-Leu-Glu-Arg-Ser (SPSLERS). To validate these observations we performed our experiments using a GSK3β knockout MEF cell line (Figure 2, Figure S1 and Figure S2) (kindly provided by Dr. James R. Woodgett of Samuel Lunenfeld Research Institute Toronto, Ontario, Canada). We made use of the parental Drosha-GFP reporter construct and two mutant constructs in which both Ser 300/302 were mutated to either a phospho-resistant or mimetic moiety. We transfected constructs individually into WT and KO MEF cells and noted their cellular distribution. As expected, WT-Drosha and phospho-mimetic Drosha behaved similarly with both localizing to the nucleus. In contrast, the phospho-resistant construct revealed a diffuse pattern similar to a construct encoding only GFP (Figure 3). These results suggested that GSK3β was indeed involved in correctly localizing Drosha to the nucleus.

### GSK3β phosphorylates Drosha

**Figure 4:**
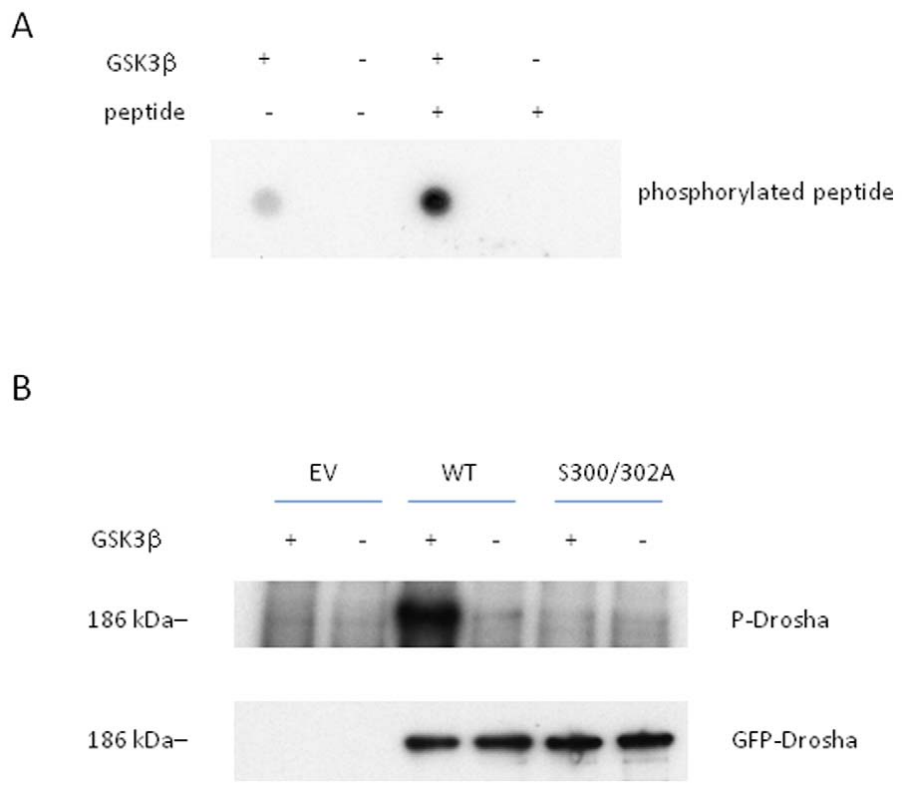
GSK3β phosphorylates Drosha at S300 and S302. *In vitro* kinase assays were performed using (A) peptide fragments of Drosha or (B) immunoprecipitated Drosha protein. **A**. A synthetic Drosha peptide (H-RERHRHRDNRRSPSLERSYKKEYKR-CONH2) was incubated with purified recombinant GSK3β enzyme for 1 hour. The reaction mixture (1 µl) was dot blotted to a nitrocellulose membrane and kinase activity was identified by x-ray film exposure. **B**. Immunoprecipitated GFP-Drosha wt or S300A/S302A was also used for *in vitro* GSK3β kinase assay with or without purified recombinant GSK3β enzyme. The reaction mixture was resolved by SDS-PAGE gel and transferred to a nitrocellulose membrane by electrophoresis before exposing to an X-ray film. The top panel shows that GFP-Drosha wt, but not GFP-Drosha S300A/S302A, was phosphorylated by GSK3β. The bottom panel shows that the expression levels of GFP-Drosha wt and S300A/S302A mutant were relatively equal in GSK3β knockout MEF cells.

**Figure 5:**
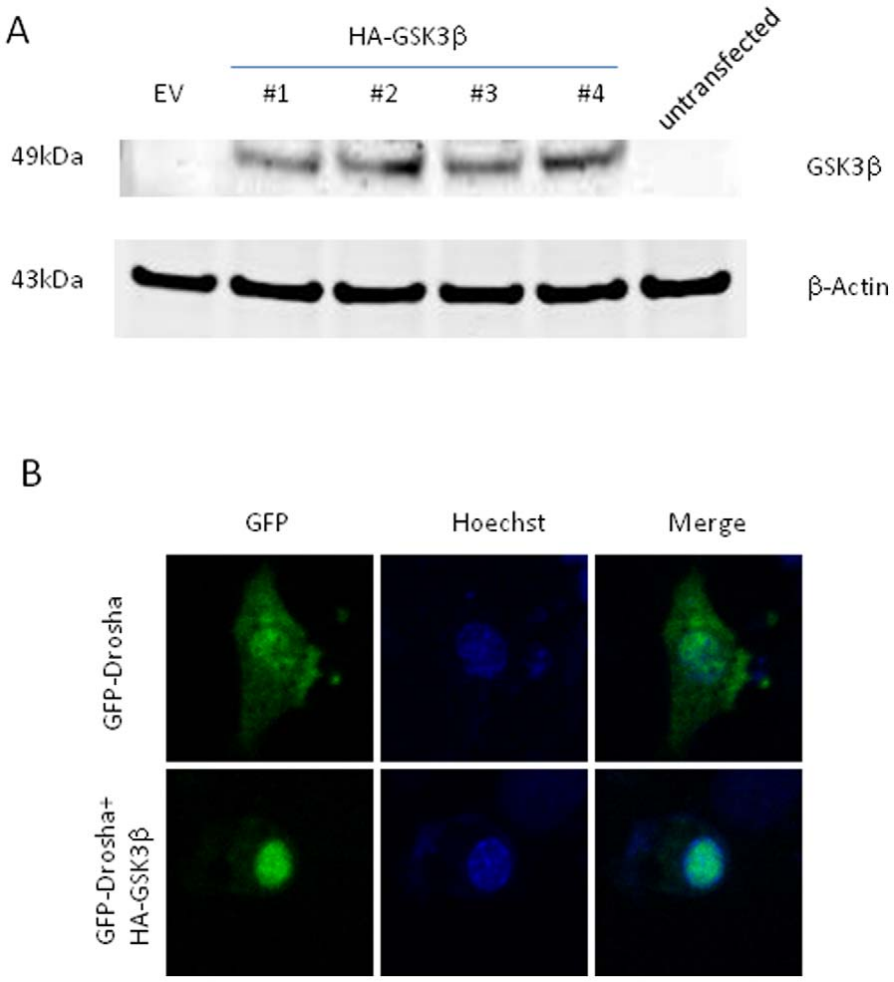
Overexpression of GSK3β in GSK3β^−/−^ MEF cells localizes Drosha to the nucleus. A. HA-GSK3β plasmid was expressed in GSK3β^−/−^ MEF cells. #1∼#4 shows protein expression by different colonies of HA-GSK3β plasmid upon cellular transfection. B. GSK3β^−/−^ MEF cells were transfected with GFP-Drosha plus empty vector or GFP-Drosha plus HA-GSK3β respectively. Confocal imaging was performed as in Figures 1 and 3 and revealed restoration of Drosha nuclear localization after add in of GSK3**β**.

To experimentally verify our bioinformatic prediction and cell culture results, we first performed an *in vitro* kinase assay using a synthetic Drosha peptide fragment harboring the putative GSK3β phosphorylation site. Dot blot analysis revealed kinase activity upon co-incubation of peptide fragment and recombinant GSK3β (Figure 4A). Previous reports have identified autophosphphorylation capacity of GSK3β and indeed we saw a faint reaction in the control lane employing enzyme only (Figure 4A). A challenge of working with Drosha is the lack of availability of pure recombinant protein ascribed to its size (∼150 kD) [20]. To circumvent this problem, we transfected GSK3β^−/−^ MEF cells with our Drosha-GFP or S300A/S302A mutant, respectively. A polyclonal antibody to GFP was used to pull down adequate amounts of protein. Importantly, both constructs expressed similar amounts of protein upon cellular transfection. As seen in Figure 4B, Figure S3 and Figure S4, only the WT construct could be phosphorylated *in vitro* by GSK3β. To further confirm that GSK3β is required for Drosha nuclear localization, a construct encoding HA-GSK3β (Addgene cat.#14753) was co-transfected with GFP-Drosha. Overexpression of GSK3β in GSK3β^−/−^ MEF cells led to complete restoration of phenotype with Drosha nuclear localization (Figure 5).

## Discussion

GSK3β is an ubiquitous kinase implicated in many processes central to cellular health [24]–[30]. Here, we show that this kinase plays a central role in localization of a protein that is intimately involved in the biogenesis of miRNA. It is important to note that while our phospho-resistant constructs did not selectively localize to the nucleus, as seen in the micrographs, a diffuse pattern was observed in which some Drosha protein was present in the nucleus. These results would seem to suggest that while GSK3β mediated phosphorylation increases nuclear localization, other mechanisms are also present, albeit less efficient, that lead to localization. Exactly how important GSK3β phosphorylation of Drosha is to the miRNA biogenesis pathway would depend upon profiling mature miRNA levels in KO cells by array. It may be that residual levels of Drosha are sufficient to process miRNA or that certain miRNA species are far better substrates for the enzyme and thereby not as sensitive to overall enzyme levels. Finally, several reports have recently identified aberrant cellular localization of Drosha with its presence in both cytoplasmic and nuclear compartments. These aberrations appear to be present in various malignancies including those of the cervix and esophagus [31],[32]. It would be interesting to gauge what role, if any, GSK3β plays in Drosha localization in these and other disease states.

Lastly, the unbridled success of imatininb has propelled the search for other kinase inhibitors in the hopes of forwarding the treatment of neoplastic disease. Indeed, many inhibitors of GSK3β are in various stages of the pre-clinical pipeline [33],[34]. Given the increasingly apparent central role that miRNA play in cellular homeostasis, our findings suggest that GSK3β inhibitors should perhaps be evaluated for their perturbations of global miRNA levels prior to their consideration as potential clinical grade reagents.

## Supporting Information

Figure S1 **Full gel of top panel of** **Figure 2** **.** GSK3β expression levels in WT and GSK3β^−/−^ MEF cells.
(TIF)

Figure S2 **Full gel of bottom panel of** **Figure 2** **.** β-actin expression in WT and GSK3β^−/−^ MEF cells.
(TIF)

Figure S3 **Full gel of top panel of** **Figure 4B** **.** GSK3β phosphorylates Drosha at Ser 300 and Ser 302.
(TIF)

Figure S4 **Full gel of bottom panel of** **Figure 4B** **.** GFP-Drosha WT and mutant protein expression patterns are equivalent.
(TIF)
