# Supplementary figures and images for: Glycogen Synthase Kinase 3 Beta (GSK3β) Phosphorylates the RNAase III Enzyme Drosha at S300 and S302
Source: PLoS One. 2011 Jun 3;6(6):e20391. doi: 10.1371/journal.pone.0020391 (PMC3108596; doi:10.1371/journal.pone.0020391)

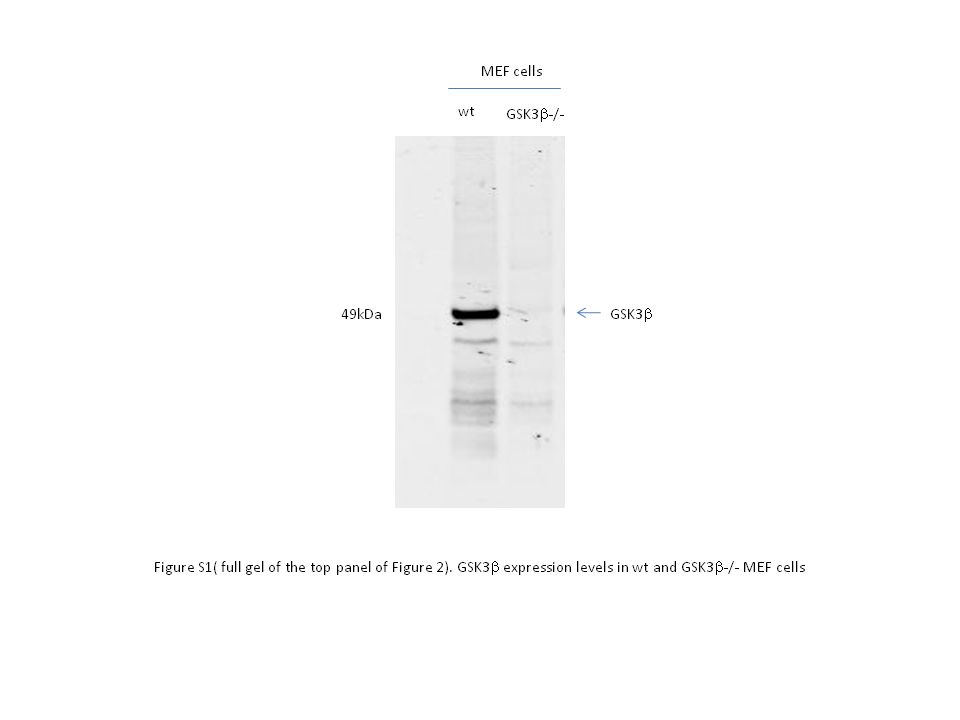

Supplement: Figure S1 — Full gel of top panel of Figure 2 . GSK3β expression levels in WT and GSK3β−/− MEF cells. (TIF) [file pone.0020391.s001.tif]

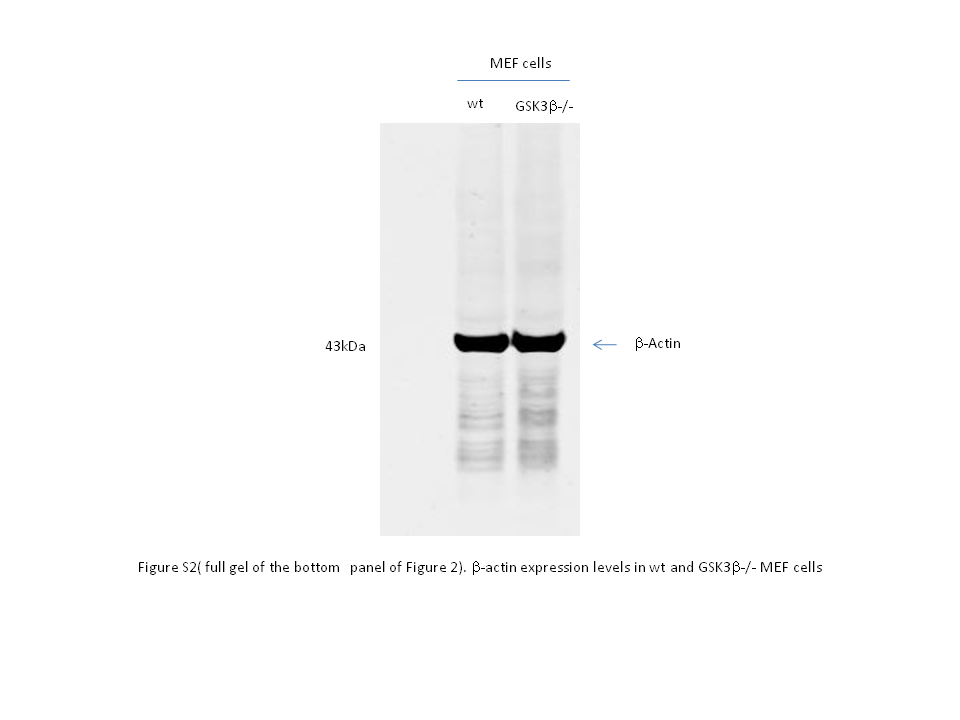

Supplement: Figure S2 — Full gel of bottom panel of Figure 2 . β-actin expression in WT and GSK3β−/− MEF cells. (TIF) [file pone.0020391.s002.tif]

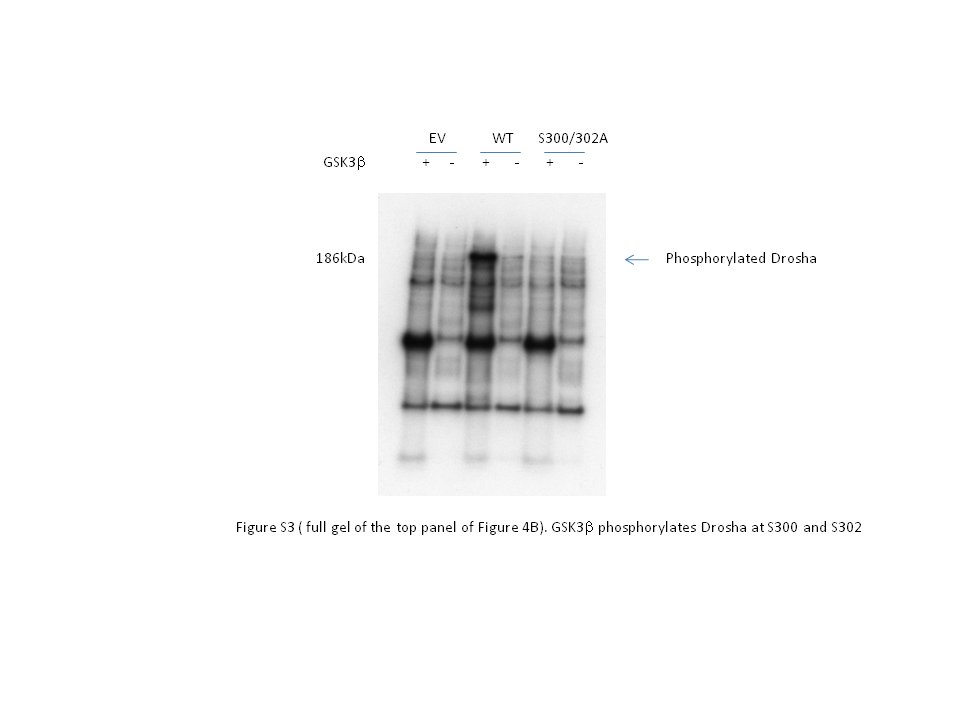

Supplement: Figure S3 — Full gel of top panel of Figure 4B . GSK3β phosphorylates Drosha at Ser 300 and Ser 302. (TIF) [file pone.0020391.s003.tif]

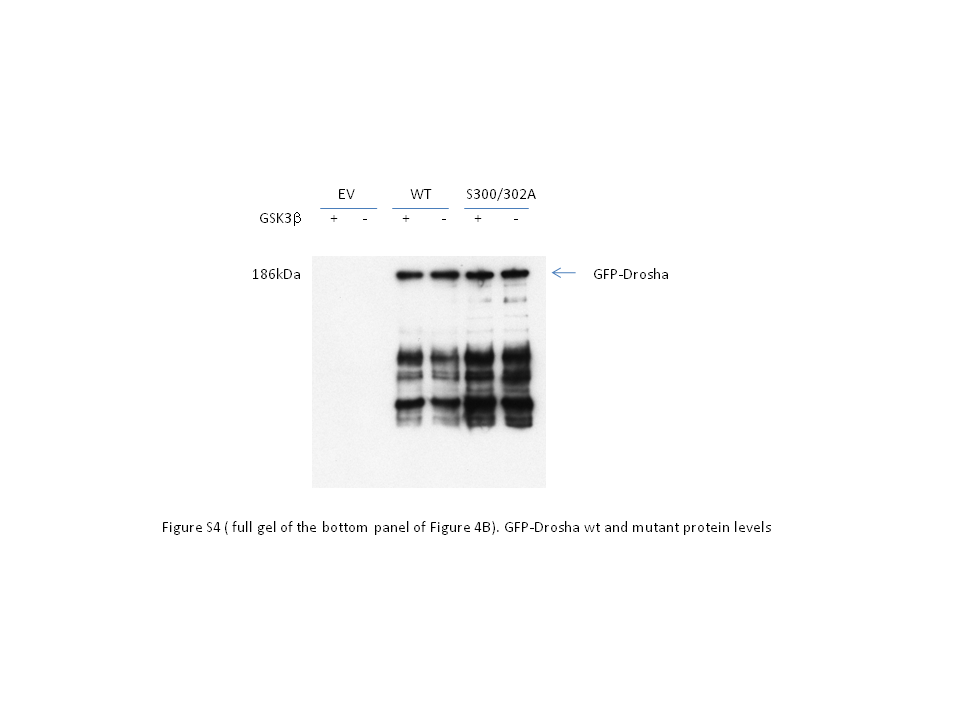

Supplement: Figure S4 — Full gel of bottom panel of Figure 4B . GFP-Drosha WT and mutant protein expression patterns are equivalent. (TIF) [file pone.0020391.s004.tif]
